# Functional Impairments and Adverse Outcomes Associated with ADHD: A Literature Review

**DOI:** 10.3390/medicina62091661

**Published:** 2026-08-29

**Authors:** Farzad Salehpour, Francisco Gonzalez-Lima

**Affiliations:** 1 Texas Consortium in Behavioral Neuroscience, Department of Psychology, The University of Texas at Austin, 108 E. Dean Keeton Stop A8000, Austin, TX 78712, USA; gonzalezlima@utexas.edu; 2 Interdisciplinary Neuroscience Program, Department of Psychiatry and Behavioral Sciences, Dell Medical School, The University of Texas at Austin, Austin, TX 78712, USA

**Keywords:** ADHD, functional impairment, psychosocial burden, medical health risks, risk-taking behaviors, mortality

## Abstract

*Background and Objectives:* Attention-deficit/hyperactivity disorder (ADHD) is increasingly recognized as a lifespan neurodevelopmental condition with far-reaching consequences for functioning and health that extend well beyond its core signs and symptoms. This narrative review synthesizes evidence on the real-world impact of ADHD across the lifespan, with a focus on academic and occupational outcomes, interpersonal and family functioning, risk-taking and behavioral outcomes, accidental and sport-related injuries, physical and medical health burdens, substance use disorders, sexual health risks, and mortality. *Materials and Methods*: A structured search of PubMed, Scopus, and Web of Science supplemented by reference-list screening was conducted to identify relevant clinical studies, epidemiological studies, longitudinal investigations, systematic reviews, and meta-analyses published between 2000 and 2025. Evidence was synthesized narratively, with greater interpretive weight given to systematic reviews and meta-analyses, large epidemiological datasets, longitudinal studies, and investigations accounting for important potential confounding factors. *Results*: Findings indicate that ADHD is associated with substantial impairments across functional, behavioral, and health domains. However, the strength and magnitude of these associations vary and may be influenced by symptom presentation, neurocognitive characteristics, psychiatric comorbidities, lifestyle behaviors, and broader clinical and contextual factors, with evidence of bidirectional relationships for some outcomes. These findings caution against uniformly interpreting adverse outcomes as direct causal consequences of ADHD and support interconnected neurocognitive, reward/motivational, emotional, and environmental pathways linking ADHD features with downstream impairment. Clinically, the evidence underscores the importance of assessing functional impairment alongside ADHD signs and symptoms. *Conclusions*: Overall, ADHD is associated with substantial and heterogeneous burdens across the lifespan, highlighting the need for multidimensional assessment, further longitudinal and mechanistic research, and timely access to evidence-based interventions.

## 1. Introduction

Attention-deficit/hyperactivity disorder (ADHD) is a common neurodevelopmental disorder characterized by developmentally inappropriate and impairing levels of inattention, hyperactivity, and impulsivity [1]. Although traditionally considered a childhood disorder, symptoms frequently persist into adolescence and adulthood, often resulting in substantial impairments across multiple domains of functioning [2,3,4]. In the clinical evaluation of ADHD, a fundamental distinction is made between objective signs, the visible behavioral markers of inattention and hyperactivity, and subjective symptoms, which encompass the individual’s internal neurocognitive and emotional struggles. While external observers may witness disorganization or restlessness, the individual often contends with debilitating, hidden challenges such as executive dysfunction, time blindness, and emotional dysregulation. In children and adolescents, signs of inattention (e.g., difficulty focusing, disorganization, forgetfulness) and hyperactivity/impulsivity (e.g., fidgeting, excessive talking, interrupting) interfere with daily activities, disrupting tasks such as homework, family routines, and peer play. Following caregiver instructions is often challenging, and youths with ADHD frequently experience conflicts at school and strained relationships within their families [5]. Adults with ADHD similarly report high levels of disability, reduced quality of life, and significant functional impairments. They commonly face real-life challenges across various domains, including academic performance, workplace productivity and occupational success, self-care and physical health, social functioning, and increased medical and mortality risks [6]. A qualitative international study has shown that adults with ADHD in the United States, Canada, the United Kingdom, France, Germany, the Netherlands, and Italy share similar narratives in major domains of functioning and exhibit similar main signs and symptoms of ADHD. This suggests that the burden and the impacts of ADHD are similar across many Western countries and are not limited to their specific socio-cultural structures and expectations for individuals [7].

A central issue in conceptualizing ADHD-related impairments concerns the definition of functional impairment itself. During the Diagnostic and Statistical Manual of Mental Disorders, 5th Edition (DSM-5), revision process, considerable debate emerged regarding whether functional impairment should remain required for diagnosing mental disorders, including ADHD [8]. Ultimately, DSM-5 retained the impairment criterion but broadened its definition so that functional difficulties only need to reduce the quality of social, academic, or occupational functioning, rather than meet the DSM-IV threshold of being clinically significant. This shift acknowledges that impairment may not always be outwardly observable. Individuals may appear functional in academic or work settings yet experience substantial internal distress, fatigue, or unsustainable compensatory effort to manage their signs and symptoms [9]. Understanding impairment through this broader lens is critical for appreciating the diverse ways ADHD disrupts everyday functioning.

Several recent reviews have synthesized the broad range of adverse outcomes and functional consequences associated with ADHD across the lifespan [4,10,11]. For example, French et al. conducted an umbrella review of 125 reviews and synthesized evidence across mental health, physical health, and social/lifestyle domains, providing one of the most comprehensive overviews of ADHD-associated outcomes currently available [4]. Building on these important syntheses, the present narrative review focuses specifically on the major domains in which ADHD contributes to functional, psychosocial, and medical burdens across the lifespan. By integrating findings from child, adolescent, and adult populations, this review aims to provide a clinically oriented perspective on the breadth and persistence of ADHD-related impairments. Particular emphasis is placed on the real-world consequences of these impairments and their implications for assessment, intervention, and future research.

## 2. Literature Search and Methodological Approach

This narrative review aims to provide a broad synthesis of the functional, psychosocial, behavioral, and medical impairments associated with ADHD across the lifespan. A structured literature search was conducted in PubMed, Scopus, and Web of Science and supplemented by manual screening of reference lists from relevant articles, systematic reviews, and meta-analyses published between January 2000 and September 2025.

Search terms included combinations of “ADHD”, “attention-deficit/hyperactivity disorder”, “functional impairment”, “academic performance”, “occupational outcomes”, “social functioning”, “relationship problems”, “injury”, “criminality”, “aggression”, “obesity”, “physical health”, “substance use disorder”, “sexual health”, and “mortality”.

Only articles published in English were considered. Articles were considered eligible when they examined individuals with ADHD or ADHD symptoms and reported outcomes relevant to at least one of the functional, psychosocial, behavioral, lifestyle, physical-health, or mortality domains addressed in this review. Studies were excluded when they did not specifically examine ADHD-related outcomes, did not provide information relevant to functional impairment or associated adverse outcomes, or fell outside the predefined scope of the review. Titles and abstracts were screened, followed by a full-text review of studies considered relevant to the objectives of this manuscript. Priority was given to systematic reviews, meta-analyses, large epidemiological studies, longitudinal investigations, and influential clinical studies addressing functional impairments associated with ADHD. Reference lists of relevant reviews and key studies were additionally examined to identify pertinent publications not captured through the database searches.

Because the objective of this article was to provide a comprehensive narrative synthesis rather than a systematic review of a narrowly defined research question, the review was not conducted according to PRISMA guidelines, and no formal quantitative synthesis or risk-of-bias assessment was performed. Instead, the evidence was synthesized narratively, with emphasis placed on the consistency, strength, and clinical relevance of findings across major domains of functioning. Greater interpretive weight was given to systematic reviews and meta-analyses, large epidemiological datasets, longitudinal studies, and investigations that accounted for important potential confounding factors. Where findings were inconsistent, differences in study design, population characteristics, psychiatric comorbidities, outcome definitions, follow-up duration, and statistical adjustment were considered when interpreting the evidence. The review therefore focused on studies considered most relevant to understanding the functional, psychosocial, behavioral, and medical burdens associated with ADHD across the lifespan.

ChatGPT (OpenAI, GPT-5) was used during manuscript preparation and revision as a writing and editorial aid to improve the language and clarity of the authors’ synthesis of the literature. All AI-assisted content was reviewed, verified, and revised by the authors, who take full responsibility for the final manuscript.

## 3. Major Domains of Impairment Associated with ADHD

### 3.1. Functional Impairments

#### 3.1.1. Poor Academic Performance

Academic outcomes are generally influenced by various factors, including the ability to learn and process information, executive functioning, organizational and time-management skills, and the social skills required for successful participation in group projects [12]. It is well established that long-term academic outcomes are adversely affected by ADHD, and poor academic performance is often the primary reason for an individual’s initial referral for clinical evaluation [13]. A longitudinal study with an 8-year follow-up found that preschool children with ADHD had poorer reading, spelling, and mathematics achievement test scores during adolescence than their peers without ADHD [14]. Surprisingly, individuals diagnosed with the inattentive presentation in childhood tend to have lower academic achievement scores in adolescence than those diagnosed with the combined presentation, which is the most common ADHD presentation [14]. In a prospective study with a mean follow-up interval of 16.1 years, male adults who were diagnosed with ADHD as children completed 2.5 years less schooling than healthy controls. Research has also shown that children with more severe ADHD symptoms, as reported by both parents and teachers, are more likely to exhibit executive function deficits that are associated with academic impairment. These findings suggest that executive dysfunction may contribute to academic difficulties in reading, writing, and mathematics among children with ADHD [15]. Some studies have suggested that inattentive symptoms and executive function deficits may be more strongly associated with academic difficulties than hyperactive–impulsive symptoms or comorbid conduct disorder (CD) [16].

#### 3.1.2. Occupational Burden

Individuals with ADHD often experience occupational challenges, including difficulties meeting work performance expectations, maintaining productivity [17], achieving occupational success [18], and sustaining long-term occupational functioning, partly due to executive function deficits and an increased vulnerability to occupational burnout [19]. ADHD symptoms, particularly inattention, have been associated with a range of work-related difficulties [20], such as being out of work and having fewer days of regular employment [21]. Inattention has been associated with procrastination and perfectionistic tendencies, while impulsivity can cause individuals to take on more tasks than they can manage. These difficulties may be further exacerbated by hyperactivity and difficulties relaxing, potentially contributing to stress and occupational burnout [22].

In a large population-based cohort study of 766,244 Scottish schoolchildren, children treated for ADHD were more likely to be unemployed after leaving school than their peers, even after adjustment for sociodemographic, perinatal, and comorbid factors (adjusted odds ratio (OR) = 1.39, 95% CI: 1.25–1.53) [23]. Attention and hyperactivity that manifested at the age of 10 years are associated with lower employment rates, poorer job quality, reduced earnings if employed, and lower overall expected earnings at the age of 30 years in both males and females [24]. In addition to the main signs of ADHD, adults with ADHD often experience emotional dysregulation and subsequent mood instability, with irritability, hot temper, and low frustration tolerance. These emotional regulation difficulties may contribute to additional workplace challenges [17]. Absences from work, often due to mental or physical health problems, are associated with an increased risk of involuntary job termination in individuals with ADHD [25]. In a study of employees from a large manufacturing firm, adults with ADHD demonstrated significantly poorer occupational functioning, including a 4–5% reduction in work performance, more than twice the odds of sickness absence (OR = 2.1, 95% CI 1.1–3.8), and twice the odds of workplace accidents and injuries (OR = 2.0, 95% CI 1.1–3.6) [26].

#### 3.1.3. Relationship Problems

Although relationship difficulties are not part of the diagnostic criteria for ADHD, the core signs and symptoms can interfere with the development of social skills, leading to challenges in various types of relationships, including those with family, peers, colleagues, and partners. Having a child with ADHD has been associated with greater family and interpersonal difficulties [27,28]. In a large European parent survey, 72% of parents reported that the parent–child relationship was negatively affected, compared with 43% in families without ADHD; impairments were also reported in sibling relationships (64% vs. 29%) and peer relationships (54% vs. 12%) [27]. Evidence shows a reciprocal relationship between the severity of a child’s ADHD signs and symptoms and the quality of the parent–child relationship [29]. Child ADHD symptoms have been associated with increased mother–child conflict and poorer parent–child interactions over time. These difficulties may contribute to the development or exacerbation of oppositional defiant disorder (ODD) and CD, which are themselves associated with further deterioration in family functioning and parent–child relationships [30]. On the other hand, inadequate parental support and dysfunctional family processes may worsen ADHD signs and symptoms [31], while good family roles and behavior control, to some extent, are associated with better-controlled ADHD signs and symptoms [32]. In families with adolescents with ADHD, affected siblings experience more impaired mother–child relationships and greater family dysfunction compared to typically developing adolescents, suggesting that greater ADHD symptom severity may be associated with more pronounced family and relationship difficulties. Affected siblings also receive less maternal affection/care, are subject to more authoritarian control, and perceive less family support than their unaffected siblings [29].

Nearly 85% of children with ADHD have difficulties in communication and social interaction [33]. As a result, children with ADHD tend to have fewer friends than their peers without ADHD [34], and almost 50% have no reciprocal friendships [35]. They are interested in friendships that are generally less intimate, cooperative, and satisfying, and more conflictual, compared to their non-ADHD peers, as they tend to value features such as entertainment and fun in their relationships [36]. Inattention may contribute to social difficulties by reducing engagement in social interactions, which can sometimes be perceived by others as withdrawal or disinterest. On the other hand, hyperactivity and impulsivity have been associated with difficulties in emotion regulation and conflict resolution, potentially contributing to misunderstandings, interpersonal conflicts, and challenges in responding to others’ emotional needs. For example, girls with ADHD, compared to typically developing girls, have been shown to experience increased difficulties in peer relationship domains such as friendship, peer interaction, social skills and functioning, and are more likely to experience peer victimization and bullying [37].

In the workplace, adults with ADHD often experience difficulties in social interactions with employers and colleagues, particularly in working as part of a team [20]. These interpersonal challenges may make it more difficult for some individuals with ADHD to function within hierarchical workplace structures and respond to supervisory feedback. Patterns of procrastination, lateness, and missed deadlines may contribute to perceptions of reduced reliability or motivation among some employees with ADHD. Feedback regarding performance difficulties may be experienced more negatively by some employees with ADHD, potentially complicating subsequent workplace interactions and motivation [38]. In the workplace, relationships with colleagues may be affected by impulsivity and emotional dysregulation. Difficulties with impulse control, interrupting others, frustration tolerance, and emotional regulation may contribute to interpersonal conflict, misunderstandings, and challenges in maintaining positive workplace relationships. Emotional dysregulation, including irritability and frustration intolerance, has been associated with workplace interpersonal difficulties, including impatience, conflict with colleagues, and occasional anger outbursts [39].

Several studies have reported associations between ADHD symptoms and difficulties in romantic relationship functioning [40,41]. For example, ADHD has been associated with lower relationship intimacy, reduced marital satisfaction [42], and higher divorce rates [43]. Emotion regulation difficulties, including poorer affect recognition, greater emotional intensity, emotional impulsivity, and increased interpersonal conflict, have been proposed as potential contributors to the association between ADHD and romantic relationship quality [40]. In a longitudinal study of young adult males, individuals with childhood ADHD reported significantly higher levels of verbal aggression and partner-directed violence than males without childhood ADHD. In unadjusted analyses, they were approximately five times more likely to report threatening to hit or throw objects at a partner (OR = 5.08), throwing objects at a partner (OR = 4.76), and hitting a partner with an object (OR = 5.85) [44]. In a large population-based study of adults in England, ADHD was associated with a moderately increased likelihood of violent behavior after adjustment for demographic characteristics, antisocial personality disorder, substance dependence, and anxiety disorders (adjusted OR = 1.75, 95% CI 1.14–2.68) [45]. Adult females with ADHD have been reported to have fewer lifetime romantic partners than females without ADHD, whereas adult males with ADHD have reported a greater number of romantic relationships than their non-ADHD counterparts [46]. Some studies have reported that males with ADHD may partner with females who report relatively lower relationship dissatisfaction, although the reasons for this finding remain unclear [41,47]. Males with the inattentive presentation of ADHD have been reported to enter stable romantic relationships at a later age than individuals with the combined presentation, who report early dating experiences similar to those of non-ADHD individuals. It has been proposed that social and interpersonal characteristics associated with predominantly inattentive symptoms may contribute to delays in initiating or establishing stable romantic relationships, although the mechanisms underlying these associations remain poorly understood [41,48].

### 3.2. Risky Behaviors

#### 3.2.1. Accidental and Sport-Related Injuries

Individuals with ADHD have an increased risk of unintentional accidental injuries such as vehicular accidents, sports injuries, and self-inflicted injuries [49,50,51].

Young adults with ADHD have been reported to receive significantly more traffic citations, particularly speeding citations, and are more likely to experience license suspension or revocation than individuals without ADHD. In one study, 20.0% of drivers with ADHD had received five or more speeding citations compared with 3.1% of controls, while 21.9% had experienced a license suspension or revocation compared with 4.7% of controls [52]. They are also more likely to lack a valid driver’s license and to engage in alcohol-impaired driving. In a prospective follow-up study to age 40, individuals with ADHD were nearly six times more likely not to possess a driver’s license than controls (OR = 5.88, 95% CI 1.95–17.7), and at age 30 they were more than five times more likely to report driving under the influence of alcohol (OR = 5.61, 95% CI 2.30–13.6) [53]. The elevated risk of accidents among individuals with ADHD has been attributed, at least in part, to external distractions (e.g., cellphone use while driving) [54] and internal distractions such as mind-wandering [55].

Research has reported a higher prevalence of ADHD among individuals with sport-related concussion [56] and mild traumatic brain injury (mTBI) [57], raising the possibility that ADHD may contribute to increased vulnerability to such injuries. A meta-analysis of five studies comprising 3023 individuals with mTBI and 9716 controls further reported a significant association between ADHD and mTBI, with a pooled relative risk of 2.0 (*z* = 6.5), indicating that ADHD and mTBI were associated at approximately twice the expected rate [58].

#### 3.2.2. Aggressive and Bullying Behaviors

Individuals with ADHD have been reported to exhibit higher levels of aggressive behaviors than their non-ADHD peers [59]. General signs of aggression may include temper tantrums, outbursts of anger, refusal to listen, and blaming others [59]. In the Multimodal Treatment Study of Children with ADHD, 54% of preadolescents with the combined presentation exhibited clinically significant aggression at baseline. Compared to the core signs and symptoms of ADHD, aggression had a much greater impact on parents’ overall impairment ratings (10% of variance vs. 2% of variance) [60]. Both types of aggression, namely proactive aggression (instrumental or purposeful aggression) and reactive aggression (impulsive aggression), are frequently observed in individuals with ADHD [61,62]. Speyer et al. [62], using a symptom-level analysis, aimed to investigate the links between ADHD inattentiveness and hyperactivity/impulsivity with aggressive behaviors (reactive and proactive aggression) in children. Hyperactivity and impulsivity were linked to reactive aggression but also to a form of proactive aggression, specifically dominating other children [62]. These dominance strategies have been proposed as one mechanism through which some children may attempt to achieve social status, particularly when self-regulatory demands make socially adaptive strategies more difficult [63]. The results showed that inattention also plays a significant role in both types of aggression. It has been suggested that inattentive symptoms may contribute to difficulties in social information processing, potentially leading children to miss important social cues and increasing the likelihood of interpersonal conflict [64]. Speyer et al. [62] suggested that, while proactive aggression is less directly connected to ADHD, inattentive and hyperactive/impulsive behaviors may drive children to use aggression instrumentally, particularly in response to peer rejection. Supporting the notion that proactive aggression is more indirectly linked to ADHD, it has also been proposed that when children with ADHD signs and symptoms are rejected by their non-ADHD peers, they may become affiliated with antisocial peers, potentially increasing exposure to and reinforcement of aggressive behaviors through a process known as peer deviancy training [65]. In a longitudinal network analysis of 1246 children assessed at ages 7, 9, and 11 years, Speyer et al. also found that inattentive symptoms showed increasingly stronger associations with aggressive behaviors over time and exerted the strongest direct and indirect influence on both reactive and proactive aggression, whereas hyperactive/impulsive symptoms tended to decline with age [62].

Conceptually, some researchers have proposed that aggression and ADHD represent distinct yet correlated dimensions of externalizing behavior [66]. Supporting this view, Waschbusch et al. [67], in a meta-analysis, reported that children with co-occurring hyperactive–impulsive–attention problems and conduct problems exhibited more severe conduct symptoms than children with hyperactive–impulsive–attention problems alone, with a large effect size (Cohen’s *d* = 1.07, 95% CI = 0.95–1.18), and than those with conduct problems alone, with a moderate-to-large effect size (*d* = 0.62, 95% CI = 0.49–0.74). These findings suggest that the co-occurrence of ADHD-related symptoms and aggressive or conduct problems is associated with greater clinical severity and psychosocial dysfunction [67]. Accordingly, reactive aggression has been proposed as a marker of greater clinical severity that may exacerbate functional impairments, rather than simply reflecting the presence of comorbid ODD or CD. In contrast, other researchers have proposed that emotional dysregulation, which Barkley conceptualizes as impulsivity expressed in the emotional domain, may partially account for the aggressive behaviors observed in individuals with ADHD [68].

In a nationally representative sample of 71,811 children and adolescents with ADHD aged 6–17 years from the US National Survey of Children’s Health, 46.9% (95% CI = 44.0–49.8%) were reported to be victims of bullying and 16.2% (95% CI = 14.1–18.5%) engaged in bullying behaviors [69], compared with corresponding prevalences of 23% and 6%, respectively, in the general pediatric population [70]. In multivariable analyses, difficulties making or keeping friends emerged as the strongest predictor of bullying victimization, with children reporting a little difficulty exhibiting 4.6-fold higher odds (95% CI = 3.49–6.09) and those reporting substantial difficulty exhibiting 16.50-fold higher odds (95% CI = 11.36–23.97) compared with children without friendship difficulties. Developmental delay or intellectual disability was also independently associated with bullying victimization (OR = 1.47, 95% CI = 1.09–1.99), while repeated school contacts regarding behavioral problems doubled the odds of victimization (OR = 2.00, 95% CI = 1.47–2.71) [69]. In a study of 1707 children and adolescents aged 6–18 years, maternal ratings indicated that children with the ADHD combined presentation exhibited substantially higher rates of bullying involvement than controls, with 62% being victims and 43% being bullies, compared with 17% and 9%, respectively, among controls. Approximately 31% of children with the combined presentation were classified as both bullies and victims, whereas the corresponding prevalence among controls was 2.7%. Children with the inattentive presentation also demonstrated elevated victimization rates (44%) but substantially lower rates of bullying perpetration (14%). After controlling for anxiety and depressive symptoms, children with the combined presentation continued to exhibit significantly higher levels of bullying victimization and perpetration than controls. However, adjustment for comorbid ODD eliminated group differences in bullying ratings, suggesting that externalizing symptoms and comorbid disruptive behaviors may contribute substantially to bullying perpetration [71].

As social context plays an important role in bullying, several mechanisms have been proposed to explain why children with ADHD may be particularly vulnerable to peer victimization. In a survey of 1315 middle-school students, children who reported taking medication for ADHD were more likely to report bullying others (13% vs. 8%) and were also more likely to report being victimized by bullies (34% vs. 22%) compared with their peers. Path analyses further indicated that the association between ADHD and bullying victimization was largely independent of self-control, whereas the association between ADHD and bullying perpetration was primarily mediated through lower self-control. Children with ADHD frequently experience poor peer status, have fewer friends, and may exhibit deficits in social skills or inappropriate behaviors that have been proposed to increase their vulnerability to bullying and peer aggression. In addition, learning difficulties and disciplinary problems may negatively affect peer perceptions and social standing among children with ADHD [72].

#### 3.2.3. Violence and Crime

In a nationally representative study of 11,238 young adults, Fang et al. [73] reported that childhood ADHD remained significantly associated with intimate partner violence resulting in injury after adjustment for CD and sociodemographic factors (OR = 1.53, 95% CI = 1.04–2.28). Individuals with ADHD alone exhibited 1.8-fold higher odds of perpetrating intimate partner violence resulting in injury (95% CI = 1.08–3.04), whereas co-occurring ADHD and CD conferred an even greater risk (OR = 3.75, 95% CI = 2.15–6.56). In contrast, CD independently predicted both non-injurious intimate partner violence (OR = 1.95, 95% CI = 1.62–2.36) and violence resulting in injury (OR = 3.20, 95% CI = 2.50–4.11), suggesting that ADHD and CD may exert both independent and combined effects on violent behavior in intimate relationships [73]. Consistent with these findings, a meta-analysis of 20 studies encompassing 48 effect-size estimates and 6261 participants found a significant overall association between ADHD and criminal or delinquent behavior (mean effect size, *Mz* = 0.155). The association was strongest for attentional deficits alone (*Mz* = 0.254), suggesting that ADHD-related symptoms constitute a meaningful risk factor for antisocial and delinquent behavior [74]. In a nationwide Danish follow-up study, 47% of children with ADHD had at least one criminal conviction by adulthood. Compared with the general population, childhood ADHD was associated with a 5.6-fold increased risk of any criminal conviction (95% CI = 5.2–6.1) and a 12-fold increased risk of violent convictions (95% CI = 9.9–14.5) [75]. A large population-based study of 7369 adults from England found that ADHD was associated with an increased likelihood of violent behavior. After adjusting for demographic factors and major psychiatric comorbidities, adults with ADHD had 75% higher odds of reporting violence in the previous five years (OR = 1.75, 95% CI = 1.14–2.68). Furthermore, hyperactivity symptoms, rather than inattention symptoms, were independently associated with violent behavior (OR = 1.15, 95% CI = 1.08–1.23), particularly violence directed toward intimate partners, family members, and friends [45].

Historically, findings have been mixed regarding whether ADHD alone, independent of comorbid CD or ODD, predicts later criminality. Several early longitudinal studies suggested that ADHD alone was not independently associated with criminal behavior in adulthood [76,77]. In a prospective 30-year follow-up study, Satterfield et al. [76] examined 179 boys with hyperactive–impulsive or combined ADHD presentations, approximately 78% of whom exhibited childhood conduct problems. Participants were followed into adulthood (mean follow-up age ~37 years), and criminal outcomes were assessed using official arrest records. Boys with ADHD and childhood conduct problems exhibited significantly higher rates of criminal justice involvement than controls, with 44.1% arrested (OR = 4.57, 95% CI = 2.20–10.28), 29.0% convicted (OR = 4.68, 95% CI = 1.88–14.03), and 26.3% incarcerated (OR = 4.08, 95% CI = 1.63–12.31). They were also more likely to experience felony arrests (38.5% vs. 13.3%; OR = 4.06, 95% CI = 1.91–9.46) and multiple felony arrests (26.0% vs. 8.0%; OR = 4.08, 95% CI = 1.63–12.25) than controls. Children with ADHD who did not exhibit childhood conduct problems had a felony recidivism rate of only 7.8%, which did not differ significantly from the control group rate of 8.0%, leading the authors to conclude that ADHD alone was not associated with an increased risk of adult criminality [76]. Consistent with Satterfield et al. [76], Mordre et al. [77] followed 541 former child psychiatric inpatients for 19–41 years using official criminal records and found that ADHD alone was not associated with an increased risk of later delinquency (27% convicted; Relative Risk, RR = 1.2, 95% CI = 0.7–2.2). In contrast, conduct disorder was associated with a significantly increased risk of adult criminality (RR = 2.0, 95% CI = 1.2–3.4), and hyperkinetic conduct disorder (ADHD combined with conduct disorder) conferred an even greater risk (RR = 2.7, 95% CI = 1.6–4.4). Based on these findings, the authors concluded that conduct problems, rather than ADHD symptoms alone, appear to account for much of the association between childhood ADHD and later criminal behavior [77].

In contrast to earlier studies, more recent longitudinal investigations have reported that ADHD remains independently associated with an elevated risk of criminality even after accounting for comorbid CD and ODD [78,79]. Using Swedish national registry data, Lundström et al. [78] examined the association between childhood neurodevelopmental disorders and subsequent violent criminality. Individuals diagnosed with ADHD had a markedly increased risk of violent criminal convictions compared with matched controls (OR = 4.6, 95% CI = 3.7–5.7). Although adjustment for parental socioeconomic factors, ODD, and CD attenuated the association, ADHD remained significantly associated with violent criminality (OR = 3.7, 95% CI = 2.9–4.9). Furthermore, even after adjustment for a broad range of psychiatric and educational factors, including schizophrenia, bipolar disorder, psychosis, substance misuse, and school failure, ADHD remained independently associated with violent criminal behavior (OR = 2.7, 95% CI = 2.0–3.8). These findings suggest that the association between ADHD and violent criminality cannot be fully explained by comorbid disruptive behavior disorders or other measured psychiatric risk factors. Elevated risks of violent offending were also observed among unaffected siblings of individuals with ADHD. Compared with matched controls, unaffected full siblings had a 50% increased risk of violent criminality (OR = 1.5, 95% CI = 1.0–2.3), whereas unaffected half-siblings had an 80% increased risk (OR = 1.8, 95% CI = 1.2–2.8). Because full and half-siblings exhibited high risks despite differing degrees of genetic relatedness, the authors suggested that shared environmental factors, such as psychosocial adversity, family conflict, and severe marital discord, in addition to familial liability, may contribute to the association between ADHD and violent criminality [78]. Furthermore, a meta-analysis and systematic review including 15,442 individuals with childhood ADHD from nine longitudinal samples found that ADHD was significantly associated with increased risks of arrest (RR = 2.2, 95% CI = 1.3–3.5), conviction (RR = 3.3, 95% CI = 2.1–5.2), and incarceration (RR = 2.9, 95% CI = 1.9–4.3) during adolescence and adulthood. The review also reported that individuals with ADHD tended to engage in antisocial behavior at a younger age, had an increased risk of criminal recidivism, and most commonly committed theft-, assault-, drug-, and weapon-related offenses [80].

Consistent with the findings of Lundström et al. [78], Mohr-Jensen et al. [79] also conducted a nationwide Danish register-based study including 4231 individuals with childhood ADHD who were followed to a mean age of 22 years. During follow-up, 32.0% of individuals with ADHD had at least one criminal conviction compared with 15.6% of controls, while 18.6% experienced incarceration compared with 6.8% of controls. Childhood ADHD was associated with a 2.4-fold increased risk of conviction (HR = 2.4, 95% CI = 2.3–2.6) and a threefold increased risk of incarceration (HR = 3.0, 95% CI = 2.8–3.3). After adjustment for psychiatric comorbidity, parental psychopathology, family composition, socioeconomic status, and other familial risk factors, ADHD remained significantly associated with conviction (HR = 1.6, 95% CI = 1.5–1.8) and incarceration (HR = 1.7, 95% CI = 1.5–1.9). Comorbid substance use disorder, ODD/CD, low family socioeconomic status, parental incarceration, and family disruption were identified as independent predictors of criminal outcomes. Almost 71% of participants received ADHD medication during follow-up, and active medication treatment was associated with a 20% reduction in conviction risk (HR = 0.8, 95% CI = 0.7–0.9) and a 30% reduction in incarceration risk (HR = 0.7, 95% CI = 0.6–0.8). Nevertheless, ADHD remained significantly associated with both conviction and incarceration after adjustment for medication use and other risk factors [79].

Discrepancies between studies regarding whether ADHD independently increases the risk of criminality may be attributable to several methodological and conceptual differences. These include variations in study design (e.g., clinical versus population-based cohorts), diagnostic approaches (e.g., DSM-based diagnoses, ICD classifications, or dimensional assessments of ADHD symptoms), and outcome measures (e.g., arrests, convictions, incarcerations, self-reported offending, or antisocial personality disorder). Additionally, studies differed substantially in how comorbid ODD and CD were assessed, controlled for, or incorporated into analyses, which may have contributed to inconsistent estimates of the association between ADHD and criminality. Differences in sample characteristics, follow-up duration, and the availability of information on familial, socioeconomic, and psychiatric risk factors may have further influenced findings. Collectively, these methodological differences complicate direct comparisons across studies and may partially explain why some investigations concluded that criminality is primarily attributable to comorbid conduct problems, whereas others reported that ADHD remained significantly associated with criminal outcomes even after adjustment for major psychiatric and familial risk factors. These findings highlight the complexity of disentangling the independent contribution of ADHD to criminal behavior and underscore the need for longitudinal studies employing standardized diagnostic criteria and comprehensive adjustment for potential confounding variables [77].

#### 3.2.4. Sexual Risk Behaviors

During adolescence, ADHD-related difficulties in inhibitory control, planning, and emotional self-regulation have been associated with increased engagement in a range of health-risk behaviors, including risky sexual behaviors [81]. Among adolescent females with ADHD, risky sexual behaviors have been reported to include inconsistent contraceptive use, multiple sexual partners, earlier sexual debut, and an increased likelihood of unplanned pregnancy [82,83]. In a sample of college students, females with ADHD reported significantly less frequent condom use than males with ADHD (Cohen’s *d* = 0.30) and were the subgroup least likely to use condoms overall [84]. In a study of 462 young adult women aged 18–30 years, 12% reported having three or more sexual partners in the previous year. Each 5-point increase in ADHD symptom severity was associated with a 15% greater likelihood of reporting three or more sexual partners after adjustment for sociodemographic factors (adjusted OR = 1.15, 95% CI = 1.01–1.30). When ADHD symptom domains were examined separately, hyperactive–impulsive symptoms remained significantly associated with having three or more sexual partners (adjusted OR = 1.37, 95% CI = 1.08–1.73), whereas inattentive symptoms were not significantly associated after adjustment [85]. This finding is consistent with evidence indicating that hyperactive–impulsive symptoms are more strongly associated with risky sexual behaviors than inattentive symptoms, although this relationship appears to be partly explained by comorbid substance use problems [86]. Females with childhood ADHD exhibited substantially higher rates of unplanned pregnancy in adulthood than females without ADHD (39.2–48.4% vs. 10.6%), corresponding to approximately 5- to 8-fold higher odds of experiencing an unplanned pregnancy (ORs = 5.4–7.9) [87]. Similarly, males with childhood ADHD were substantially more likely to report having impregnated a partner by young adulthood than males without ADHD (24% vs. 5%; OR = 6.31). Although childhood conduct problems contributed to this association, the authors concluded that ADHD also exerted an independent influence on later risky sexual behavior [88].

Alcohol use, binge drinking, and cannabis use have also been associated with greater sexual health risks among individuals with ADHD and may partially explain the relationship between ADHD symptoms and risky sexual behaviors. In a nationwide sample of 36,236 sexually active college students, individuals with ADHD reported a greater number of sexual partners (adjusted odds ratio, aOR = 1.27), lower rates of condom use (aOR = 0.77), higher rates of condomless sex while drinking (aOR = 1.52), more sexually transmitted infections (aOR = 1.29), and a greater likelihood of unplanned pregnancy (aOR = 1.72) than their peers without ADHD. Alcohol use, binge drinking, and cannabis use significantly moderated several of these associations, indicating that substance use may amplify sexual health risks among college students with ADHD [89]. Furthermore, substance use disorders, which are common among individuals with ADHD, have been associated with risky sexual behaviors, including sexual activity with unfamiliar partners and sex exchanged for drugs, potentially increasing the risk of unintended pregnancy [90]. Family factors may also contribute, as insufficient parental monitoring, inconsistent rule enforcement, and lower-quality family-based sexual education have been associated with earlier sexual initiation and less consistent use of condoms and contraceptives among adolescents [90].

### 3.3. Physical and Medical Health Burdens

#### 3.3.1. Lack of Physical Activity

Several studies have reported lower levels of physical activity among individuals with ADHD [91]. In a large population-based study of 34,675 U.S. children aged 6–17 years, children with ADHD had 21% lower odds of meeting the American Academy of Pediatrics recommendation of at least 60 min of daily physical activity than children without ADHD (aOR = 0.79, 95% CI = 0.67–0.93). Female sex was also independently associated with lower odds of achieving daily physical activity recommendations (aOR = 0.58, 95% CI = 0.51–0.66), suggesting that girls with ADHD may be particularly vulnerable to physical inactivity and could benefit from targeted interventions aimed at increasing physical activity participation [92].

Epidemiological data from a nationally representative sample of U.S. children aged 6–17 years similarly showed that children with ADHD were less likely to engage in vigorous physical activity and organized sports than their peers without ADHD, regardless of medication status or sex [93]. Among children not receiving ADHD medication, boys with ADHD had 45% higher odds of engaging in low levels of physical activity (<3 days/week; OR = 1.45, 95% CI = 1.23–1.70), whereas girls had 71% higher odds (OR = 1.71, 95% CI = 1.28–2.27). Likewise, boys and girls with ADHD were significantly less likely to participate in organized sports, with OR of 1.50 (95% CI = 1.23–1.82) and 1.39 (95% CI = 1.02–1.90), respectively, for non-participation in organized sports relative to their non-ADHD peers [93].

One longitudinal population-based study provided evidence of a bidirectional association between ADHD symptoms and physical inactivity. After adjustment for potential confounders, children with ADHD symptoms at age 8 had 60% higher odds of being physically inactive at age 16 (OR = 1.60, 95% CI = 1.20–2.13), whereas reduced physically active play at age 8 was associated with 61% higher odds of inattentive symptoms at age 16 (OR = 1.61, 95% CI = 1.16–2.24). These findings suggest reciprocal longitudinal associations between ADHD symptoms and physical inactivity across development [94].

Several factors have been proposed to explain the lower levels of physical activity observed among individuals with ADHD. Executive dysfunction, including difficulties with planning, organization, forgetfulness, sustained attention, and time management, may hinder the initiation and maintenance of regular physical activity. Hyperfocus may also act as a barrier when attention becomes intensely directed toward non-physical tasks. In addition, low self-esteem, social difficulties, reduced motivation to initiate exercise, and limited awareness of the potential benefits of physical activity have all been identified as barriers to physical activity participation among adults with ADHD [95].

Research on motor development and physical performance provides additional insight into factors that may be associated with lower levels of physical activity among individuals with ADHD. Low gross motor performance, delayed motor development, and poorer physical fitness have been consistently reported among children with ADHD [96]. Children with ADHD have also been reported to demonstrate lower levels of movement-skill knowledge than their peers [97]. In addition, developmental coordination disorder frequently co-occurs with ADHD and may further impair participation in physical activities. Research in adults with ADHD has likewise demonstrated lower maximal oxygen uptake (VO_2_max) than that observed in the general population, suggesting reduced aerobic fitness [98]. Collectively, these motor, coordination, and fitness-related difficulties may represent additional barriers to participation in regular physical activity among individuals with ADHD [99].

#### 3.3.2. Sedentary Behaviors

Sedentary behavior has been consistently associated with ADHD and represents another important lifestyle factor linked to the disorder. Sedentary behaviors are waking activities performed while sitting, reclining, or lying down that require very low energy expenditure (≤1.5 metabolic equivalents [METs]). One MET corresponds to the energy expenditure of sitting quietly and serves as a reference value for estimating the intensity of physical activities. Sedentary behaviors can be broadly categorized as screen-based or non-screen-based. Screen-based sedentary behaviors include television viewing; video streaming; computer, tablet, and smartphone use; and video gaming, excluding physically active electronic games. Non-screen-based sedentary behaviors include activities such as reading, seated hobbies (e.g., painting, knitting, or crafting), social interactions while seated, and sedentary transportation [100].

In a study of 424 children aged 9–11 years, elevated teacher-rated hyperactivity/inattention symptoms were independently associated with greater sedentary behavior after adjustment for potential confounders (OR = 1.13, 95% CI = 1.05–1.21) [101]. In a study of 913 adolescents aged 13–17 years, screen-based sedentary behaviors were significantly associated with overall ADHD symptoms and both inattentive and hyperactive/impulsive symptom dimensions after adjustment for age, sex, physical activity, and BMI, whereas non-screen-based sedentary behaviors showed no significant associations [102]. Meta-analytic evidence further indicates a significant association between electronic media use and ADHD symptoms, with individuals exposed to higher levels of electronic media use exhibiting approximately 2.6-fold greater odds of ADHD symptoms [103]. Several mechanisms have been proposed to explain this association. First, the high stimulus density and intensity of screen-based media, including television programs and video games, may contribute to difficulties sustaining attention in less stimulating real-world environments such as classrooms [104,105]. Second, higher BMI has been proposed as a potential mediator of the association between ADHD symptoms and excessive screen time [106].

Findings from longitudinal studies suggest that ADHD symptoms and sedentary behaviors may influence one another over time [107]. For example, higher levels of screen-based sedentary behavior have been associated with increased hyperactive and inattentive symptoms at follow-up assessments conducted one to two years later [105,108]. Similarly, greater television viewing during childhood has been associated with higher levels of inattentive symptoms during adolescence [109]. More recent evidence further suggests that greater television viewing and mobile phone use may be associated with an increased likelihood of ADHD symptoms [110]. Consequently, future longitudinal and interventional studies are needed to determine whether reducing sedentary behaviors can improve ADHD symptoms or modify their developmental trajectory.

#### 3.3.3. Poor Diet and Nutritional Habits

Impulsivity, a core feature of ADHD, has been associated with poorer dietary choices [111], while unhealthy dietary patterns have also been proposed as potential contributors to ADHD symptoms and diagnosis [112,113]. Low-quality diets characterized by frequent consumption of sweetened beverages, snacks, confectionery products, and excess caloric intake have been consistently reported among children with ADHD [114]. Adherence to a Western dietary pattern, characterized by high consumption of processed foods rich in saturated fats, salt, and refined sugars, has been associated with increased ADHD symptomatology [115]. For example, adolescents with greater adherence to a Western dietary pattern were more than twice as likely to have an ADHD diagnosis at age 14 after adjustment for potential confounders (OR = 2.21, 95% CI = 1.18–4.13) [116]. Lower adherence to a Mediterranean diet was associated with a sevenfold greater likelihood of ADHD diagnosis (OR = 7.07, 95% CI = 2.65–18.84), even after adjustment for multiple potential confounders. Additionally, higher frequencies of skipping breakfast, eating at fast-food restaurants, and consuming sugar, candy, cola beverages, and non-cola soft drinks, together with lower consumption of fatty fish, were associated with ADHD diagnosis [117].

Several studies have also reported associations between ADHD and deficiencies in iron [118], vitamin D [119], omega-3 fatty acids [120], and trace elements such as copper and zinc [121]. Dietary additives, including artificial food colorings and sodium benzoate preservatives, have likewise been implicated in the expression of ADHD symptoms both among individuals with ADHD [122] and within the general population [123]. Although these findings do not establish a causal relationship between diet and ADHD, they suggest that dietary quality and nutritional status may contribute to symptom expression and potentially influence ADHD risk, warranting further prospective and interventional research.

#### 3.3.4. Obesity

Meta-analytic evidence from 42 studies involving 48,161 individuals with ADHD demonstrated a significant association between ADHD and obesity. Children and adolescents with ADHD had 20% greater odds of obesity (OR = 1.20, 95% CI = 1.05–1.37), whereas adults with ADHD had 55% greater odds (OR = 1.55, 95% CI = 1.32–1.81) compared with individuals without ADHD [124]. Consistent with these findings, another meta-analysis of 43 studies involving 703,937 participants reported a small but significant overall association between ADHD and obesity (OR = 1.22, 95% CI = 1.11–1.34), with a weaker association in youth (OR = 1.13, 95% CI = 1.00–1.27) and a stronger association in adults (OR = 1.37, 95% CI = 1.19–1.58) [125].

Epidemiological studies have consistently reported higher rates of obesity among individuals with ADHD. A meta-analysis found that obesity prevalence was approximately 40% higher among children and adolescents with ADHD than among controls (10.3% vs. 7.4%) and approximately 70% higher among adults with ADHD (28.2% vs. 16.4%) [124]. Similar patterns were reported by Li et al., who found obesity prevalence rates of approximately 14% in children aged 6–12 years and 19% in adults with ADHD [126].

In a large longitudinal population-based cohort, Khalife et al. [94] found that children with clinically significant ADHD symptoms at age 8 had nearly twice the odds of obesity at age 16 (OR = 1.91, 95% CI = 1.10–3.33), whereas obesity did not predict later ADHD symptoms. Both inattentive and hyperactive symptoms were associated with later obesity-related outcomes, suggesting that ADHD symptoms may contribute to obesity risk over time [94]. Karhunen et al. [127] reported evidence from Mendelian randomization and polygenic risk score analyses supporting a bidirectional relationship between ADHD and obesity. Their findings suggested that shared genetic liability, together with prenatal exposure to maternal overweight or obesity, may contribute to the observed association between ADHD and obesity-related outcomes [127]. Additionally, a systematic review reported that emotion dysregulation and negative affectivity may mediate the association between ADHD symptoms and disordered eating behaviors, including loss-of-control eating, emotional overeating, binge eating, and excessive food preoccupation, which may subsequently contribute to obesity risk [128].

Karhunen et al. [127] were among the first to investigate whether prenatal exposure to maternal obesity contributes to ADHD symptoms in offspring while accounting for direct genetic influences. Using Mendelian randomization, polygenic risk score, and longitudinal cohort analyses, they found evidence that the co-occurrence of ADHD and obesity has both genetic and prenatal environmental origins. Their analyses demonstrated shared genetic liability between ADHD symptoms and BMI, suggesting a partially shared genetic etiology underlying both ADHD and obesity-related traits. Furthermore, maternal pre-pregnancy BMI remained significantly associated with offspring ADHD symptoms after adjustment for genetic risk, supporting an independent prenatal environmental contribution [127]. Others have also suggested that BMI-related genes, particularly those involved in brain reward pathways, may contribute to ADHD symptoms and represent a potential biological mechanism linking ADHD and obesity [129]. This hypothesis is further supported by evidence suggesting that, similar to other immediately rewarding behaviors, overeating may function as a form of self-medication for dopaminergic dysfunction associated with ADHD pathophysiology [130].

#### 3.3.5. Medical Health Issues

ADHD is associated with a wide range of physical health complaints and chronic medical conditions [131,132]. A prospective U.S. cohort study with more than 10 years of follow-up [133] and a retrospective analysis of U.S. healthcare claims [134] consistently reported that adults with ADHD have a higher prevalence of physical comorbidities and greater utilization of non-psychiatric healthcare services than individuals without ADHD. However, the extent to which ADHD medications contribute to these physical health outcomes remains uncertain and requires further investigation. Common adverse effects of stimulant medications, such as methylphenidate and amphetamine formulations, include insomnia, headache, abdominal pain, irritability, and modest increases in heart rate and blood pressure [135].

Meta-analytic evidence indicates that individuals with ADHD are approximately twice as likely to experience headaches as those without ADHD (OR = 2.01, 95% CI = 1.63–2.46), with a pooled headache prevalence of 26.6% among children and adolescents with ADHD. The association remained significant after adjustment for potential confounders (adjusted OR = 1.98, 95% CI = 1.60–2.45) [136]. Similarly, another meta-analysis found that ADHD was associated with 32% greater odds of migraine (OR = 1.32, 95% CI = 1.02–1.72), whereas no significant association was observed for tension-type headache [137]. Beyond headache disorders, chronic pain is also common among individuals with ADHD. Among 216 adolescents with ADHD, 66% reported chronic pain, 55% reported chronic musculoskeletal pain, and 31% experienced multisite pain [138]. In a case-control study of 154 adults with ADHD and 262 healthy controls, fibromyalgia syndrome was diagnosed in 24.7% of individuals with ADHD, whereas none of the controls met diagnostic criteria for fibromyalgia [139]. Shared dopaminergic dysfunction has been proposed as a potential mechanism linking ADHD and fibromyalgia [140]. In addition, gastrointestinal pain disorders, including aerophagia and functional abdominal pain syndrome, have been reported more frequently in children with ADHD [141].

Meta-analytic evidence indicates that adults with ADHD have a 73% higher risk of cardiovascular disease (OR = 1.73, 95% CI = 1.14–2.62), with particularly elevated risks of cardiac arrest, hemorrhagic stroke, and peripheral vascular disease/arteriosclerosis [142]. Research also suggests that the observed association between ADHD symptoms and hypertension is likely explained by obesity and related health behaviors rather than a direct effect of ADHD itself [143].

A recent systematic review and meta-analysis including 5.7 million participants found that individuals with ADHD had more than twice the odds of developing type 2 diabetes (OR = 2.29, 95% CI: 1.48–3.55). In a complementary Swedish population-based sibling study, ADHD was associated with a 2.35-fold increased risk of type 2 diabetes (HR = 2.35, 95% CI: 2.14–2.58); however, this association was markedly attenuated after adjustment for psychiatric comorbidities, indicating that substance use disorder, depression, and anxiety account for a substantial proportion of the observed relationship [144].

A population-based study found a strong trend toward a higher incidence of epilepsy among children with ADHD (RR = 2.7, 95% CI = 0.94–7.76), with affected children exhibiting earlier seizure onset and more severe epilepsy than controls [145].

Sleep disturbances are highly prevalent among individuals with ADHD across the lifespan. Children with ADHD show increased nocturnal movements [146], and more frequent periodic limb movements [147], along with higher apnea indices [146]. Meta-analytic evidence further indicates a moderate association between ADHD symptoms and sleep-disordered breathing (Hedges’ g = 0.57, 95% CI = 0.36–0.78), with ADHD symptoms showing significant improvement following adenotonsillectomy (Hedges’ g = 0.43, 95% CI = 0.30–0.55) [148]. Children with ADHD also exhibit poorer sleep quality characterized by sleep fragmentation, reduced sleep efficiency, and excessive daytime sleepiness [149]. Similarly, a meta-analysis of 13 studies found that adults with ADHD reported significantly longer sleep-onset latency, poorer sleep quality, greater daytime sleepiness, and more frequent night awakenings, while objective actigraphy demonstrated prolonged sleep-onset latency and reduced sleep efficiency compared with non-ADHD controls [150]. The relationship between sleep disturbances and ADHD appears to be bidirectional, whereby poor sleep exacerbates ADHD symptoms while ADHD-related difficulties further contribute to sleep disruption [151].

A population-based study reported markedly elevated rates of non-suicidal self-injury (69% vs. 32%) and suicidal ideation (57% vs. 28%) among adolescents with ADHD compared with controls. Importantly, ADHD remained independently associated with suicidal ideation after adjustment for childhood behavioral problems, psychiatric comorbidities, and family and social factors (adjusted OR = 6.11, 95% CI = 2.34–16.0) [152]. In terms of suicidal outcomes requiring medical attention, meta-analytic evidence indicates substantially increased risks among individuals with ADHD, including 2.37-fold higher odds of suicide attempts (95% CI: 1.64–3.43), 3.53-fold higher odds of suicidal ideation (95% CI: 2.94–4.25), 4.54-fold higher odds of suicidal plans (95% CI: 2.46–8.37), and 6.69-fold higher odds of completed suicide (95% CI: 3.24–13.79). Associations with suicide attempts and suicidal ideation remained significant after adjustment for potential confounders [153]. Studies have shown that females with ADHD exhibit higher rates of non-suicidal self-injury [154], suicidal ideation [155], suicidal behaviors (Fitzgerald, Dalsgaard, Nordentoft, & Erlangsen, 2019), and depression and suicide attempts [156] compared with males with ADHD. Longitudinal research has further shown that, by early adulthood, girls diagnosed in childhood with the combined presentation of ADHD had markedly elevated rates of non-suicidal self-injury (51%) and suicide attempts (22%) compared with those with the inattentive presentation (29% and 8%, respectively) and healthy controls (19% and 6%, respectively). The odds of self-injury and suicide attempts were 4.4-fold and 4.5-fold higher, respectively, in the combined presentation group than in controls. Importantly, these associations remained significant after adjustment for demographic factors, childhood IQ, psychiatric comorbidities, and medication status [157].

#### 3.3.6. Substance Use Disorders

ADHD substantially increases the risk of developing substance use disorders (SUDs), even in the absence of other psychiatric comorbidities [158]. In a large population-based study, both males and females with ADHD exhibited approximately fourfold higher rates of any SUDs than individuals without ADHD (males: 15.9% vs. 4.1%, adjusted HR = 4.1, 95% CI = 3.9–4.2; females: 14.4% vs. 3.2%, adjusted HR = 4.5, 95% CI = 4.3–4.7). Males with ADHD generally exhibited higher prevalence of most specific SUD subtypes than females, particularly cannabis-, sedative-, stimulant-, and multiple-substance-related disorders [159]. This difference may partly reflect that females are often diagnosed with ADHD later than males [160,161,162], potentially allowing SUDs to co-develop or coexist before ADHD is diagnosed [159]. The association between ADHD and SUDs appears to be bidirectional. A meta-analysis found that ADHD was present in approximately one in four adolescents with SUDs (25.3%, 95% CI = 20.0–31.4%) and one in five adults with SUDs (21.0%, 95% CI = 15.9–27.2%), with an overall pooled prevalence of 23.1% (95% CI = 19.4–27.2%) across individuals with SUDs [163].

Meta-analytic evidence indicates that childhood ADHD is associated with increased risks of alcohol use disorders (OR = 1.35, 95% CI = 1.11–1.64), nicotine use (OR = 2.36, 95% CI = 1.71–3.27), non-alcohol drug use disorders (OR = 3.48, 95% CI = 1.80–6.73), and cannabis use disorders (OR = 1.51, 95% CI = 1.02–2.24) later in life [164]. Consistent with these findings, a subsequent meta-analysis reported that childhood ADHD was associated with significantly increased risks of nicotine dependence (OR = 2.82, 95% CI = 2.41–3.29), alcohol abuse/dependence (OR = 1.74, 95% CI = 1.38–2.20), marijuana abuse/dependence (OR = 1.58, 95% CI = 1.16–2.14), and cocaine abuse/dependence (OR = 2.05, 95% CI = 1.38–3.04) later in life [165]. A 10-year prospective study further demonstrated that childhood ADHD predicted an increased risk of later SUDs, particularly drug use disorders and cigarette smoking, and was associated with earlier initiation of substance-related problems [166].

Impulsivity is considered a key neurobehavioral mechanism linking ADHD to SUDs [167]. An imbalance between prefrontal cognitive control networks and subcortical reward-processing circuits may reduce inhibitory control and increase vulnerability to risky behaviors, including drug experimentation [168,169].

#### 3.3.7. Sexual Health Complications

ADHD is associated with an increased risk of sexually transmitted infections (STIs) [170]. Research indicates that the prevalence of STIs is higher among adults with childhood ADHD than among matched controls (15% vs. 7%) [171]. Individuals with ADHD also develop sexually transmitted infections, including HIV, syphilis, genital warts, gonorrhea, chlamydial infection, and trichomoniasis, at an earlier age than non-ADHD controls (20.5 vs. 21.9 years) [172]. Women with ADHD have a significantly increased risk of acquiring high-risk human papillomavirus (HPV) infection (HR = 1.69, 95% CI = 1.35–2.12). The association remained significant after adjustment for age, educational level, HPV vaccination status, and maternal history of cervical neoplasia (adjusted HR = 1.53, 95% CI = 1.20–1.95) [173]. The association between ADHD and STIs is thought to be mediated by risky sexual behaviors, including earlier sexual debut, a greater number of sexual partners, less frequent condom use, and sexual activity under the influence of alcohol or drugs, the latter of which may be related to comorbid SUDs [174,175].

#### 3.3.8. Mortality Rate

Evidence from longitudinal and population-based studies indicates that ADHD is associated with substantially elevated mortality and reduced life expectancy. In a Danish nationwide cohort study with up to 32 years of follow-up, individuals with ADHD had more than twice the mortality rate of controls (5.85 vs. 2.21 deaths per 10,000 person-years), corresponding to a fully adjusted mortality rate ratio (MRR) of 2.07 (95% CI = 1.70–2.50). The excess mortality was primarily attributable to unnatural causes, particularly accidents (adjusted MRR = 2.40, 95% CI = 1.81–3.13) [176]. Using a longitudinal cohort of children with ADHD followed into adulthood, Barkley and Fischer estimated that childhood ADHD was associated with an 8.4-year reduction in total life expectancy, while persistent ADHD was associated with an estimated 12.7-year reduction in life expectancy relative to controls [177]. More recently, a UK matched-cohort study estimated that male adults with diagnosed ADHD lost an average of 6.8 years of life expectancy (95% CI = 4.50–9.11) and female adults with diagnosed ADHD an average of 8.6 years (95% CI = 6.55–10.91) compared with the general population [178].

## 4. Critical Synthesis of the Evidence

Despite the consistency of associations between ADHD and adverse outcomes across multiple functional, psychosocial, behavioral, and health domains (Table 1), the strength of the evidence supporting these associations varies considerably. Much of the available literature is observational, and differences across studies in ADHD ascertainment, sample characteristics, age and sex distributions, outcome definitions, follow-up duration, and statistical adjustment complicate direct comparisons and may contribute to inconsistent findings.

Across the major domains of functional impairment, the evidence consistently indicates adverse outcomes associated with ADHD, although the magnitude and expression of these impairments vary according to symptom presentation, neurocognitive characteristics, and other clinical and contextual factors. Within academic functioning, impairment may vary according to symptom presentation and underlying neurocognitive difficulties, with inattentive symptoms and executive dysfunction appearing more strongly associated with academic difficulties than hyperactive–impulsive symptoms or comorbid conduct problems. Occupational disadvantage is likewise consistently reported, but its expression varies across unemployment, reduced productivity and earnings, sickness absence, and workplace accidents, with inattention, executive dysfunction, emotional dysregulation, and co-occurring health problems potentially contributing to these outcomes. Relationship difficulties associated with ADHD appear particularly heterogeneous across family, peer, and romantic contexts, with evidence suggesting bidirectional influences between ADHD symptoms and interpersonal functioning, as well as variation according to sex and ADHD presentation.

A similar pattern of heterogeneity is evident across risky behavioral outcomes, where associations with ADHD appear to be influenced by symptom dimensions, psychiatric comorbidities, and contextual factors. Evidence consistently links ADHD with increased accidental and driving-related injury risk; however, the extent to which this reflects ADHD itself or associated risk behaviors, such as distraction and alcohol-impaired driving, remains difficult to disentangle, while the direction of the association between ADHD and traumatic brain injury is not fully established. Findings on aggression and bullying suggest that risk varies according to ADHD symptom dimensions and comorbid externalizing problems, with inattentive and hyperactive–impulsive symptoms showing different patterns of association with aggressive behaviors and some bullying differences becoming nonsignificant after adjustment for ODD. The relationship between ADHD and criminality appears particularly sensitive to comorbid and contextual factors, as some studies suggest that much of the elevated risk is attributable to conduct problems and familial or socioeconomic adversity, whereas others report that ADHD remains independently associated with criminal outcomes after extensive adjustment for these factors. Associations between ADHD and risky sexual behaviors also vary by symptom dimension and comorbidity, with hyperactive–impulsive symptoms appearing more strongly related to sexual risk than inattentive symptoms and some of this association potentially being explained by co-occurring substance use.

Health-related outcomes show comparable complexity, particularly regarding directionality and the contribution of behavioral and clinical factors. Longitudinal evidence suggests reciprocal relationships between ADHD symptoms and physical inactivity, while associations with sedentary behavior appear stronger for screen-based than non-screen activities. Evidence regarding obesity is similarly complex, with longitudinal findings suggesting that ADHD symptoms may precede later obesity, whereas genetic analyses support a bidirectional relationship. More broadly, psychiatric comorbidities, medication exposure, lifestyle behaviors, and other health-related factors may influence the magnitude of associations between ADHD and physical or medical outcomes.

In sum, although the available evidence strengthens confidence in several of the observed associations, the existing variations underscore the difficulty of establishing causal relationships between ADHD and many of the adverse outcomes reviewed. Accordingly, these adverse outcomes should not be viewed in isolation or interpreted as uniformly reflecting direct causal effects of the disorder. Rather, the evidence points to interconnected pathways through which core ADHD features may contribute to broader impairments across the lifespan. Therefore, greater methodological consistency and more comprehensive adjustment for relevant confounding factors are needed to clarify which adverse outcomes are more directly associated with ADHD and which arise through interacting biological, psychological, and environmental pathways. For example, neurocognitive difficulties, including deficits in sustained attention, executive dysfunction, and impaired inhibitory control, together with alterations in reward and motivational processing and emotional dysregulation, may represent intermediate mechanisms linking ADHD signs and symptoms with functional, behavioral, and health-related outcomes [179]. These mechanisms may operate across multiple domains simultaneously, helping to explain why difficulties in academic, occupational, interpersonal, behavioral, and health functioning frequently co-occur in individuals with ADHD.

## 5. Clinical Implications for ADHD Assessment and Diagnosis

Although the characteristic signs and symptoms of ADHD constitute the core diagnostic features of the disorder, their clinical significance must be considered together with the extent of functional impairment [180]. The broad and heterogeneous adverse outcomes reviewed in this paper further demonstrate that symptom presentation alone may not fully capture the functional burden experienced by individuals with ADHD and underscore the importance of assessing functional impairment in addition to ADHD signs and symptoms [181]. There was considerable debate within the DSM-5 ADHD workgroup regarding how to define severity and the level of impairment associated with ADHD. The workgroup ultimately concluded that clinicians should apply their professional judgment, integrating sign and symptom counts with the extent of functional impairment observed in daily life. This approach may improve diagnostic accuracy, particularly among individuals whose symptoms are less overt, but whose functional impairments are substantial [182]. However, two important considerations warrant particular attention when applying this principle in clinical practice.

First, clinicians should recognize that signs, symptoms, and impairments are related but distinct constructs [183]. The signs of a disorder refer to the observable behavioral manifestations associated with the condition. In the case of ADHD, the core signs (e.g., inattention, distractibility, impulsive responding, and hyperactivity) are actionable and behavioral expressions that reflect the underlying disorder. In contrast, impairments refer to the consequences that arise across various life domains (e.g., academic, occupational, social) because of these behaviors. For example, excessive distractibility while driving is considered an ADHD sign, as it reflects an abnormal cognitive–behavioral pattern. However, involvement in a traffic accident, being cited for a traffic offense, sustaining injuries, or incurring financial losses are functional impairments resulting from the persistent manifestation of excessive distractibility [184].

Second, clinical research underscores the importance of assessing functional impairment independently of signs and symptoms to improve diagnostic accuracy and prevent overestimation of ADHD prevalence [185]. For example, it has been shown that when using only the Swanson, Nolan, and Pelham IV (SNAP-IV) maternal rating scale and DSM-IV-TR criteria, ~80% of a certain sample is identified as having ADHD. When the SNAP-IV and the Child Behavior Checklist (CBCL) rating scales were used together, this percentage decreased to 60%. Surprisingly, when the Global Impairment Index (GII) was also considered along with those two measures, this percentage dropped to ~20% of the sample [186]. This result is consistent with findings from a separate study showing that failure to incorporate functional interference into the diagnostic process leads to an inflated prevalence rate of ADHD [187]. Signs, symptoms, and impairments associated with ADHD can be evaluated using multiple sources, including tallies derived from standardized assessments such as the Adult ADHD Self-Report Scale (ASRS), and validated measures of functional impairment such as the Barkley Functional Impairment Scale (BFIS) and the Weiss Functional Impairment Rating Scale (WFIRS) [188].

## 6. Limitations

This literature review has several limitations that should be considered when interpreting its findings. First, as a narrative review addressing a broad range of functional, behavioral, and health outcomes, it was not conducted according to PRISMA guidelines and did not include a formal risk-of-bias assessment or quantitative synthesis. Accordingly, the study-selection process may be less reproducible than that of a systematic review, and the relative quality of individual studies could not be formally quantified. Second, only English-language publications were considered, which may have excluded relevant evidence published in other languages. Third, substantial heterogeneity across studies in ADHD ascertainment, sample characteristics, outcome definitions, follow-up duration, and adjustment for potential confounding factors limits direct comparison across findings. Finally, the broad scope of this review necessarily limits the depth with which evidence within each individual outcome domain could be evaluated, highlighting the need for future systematic reviews and meta-analyses focused on specific domains to provide more structured and quantitative evaluation of the evidence.

## 7. Conclusions and Future Directions

**Figure 1:**
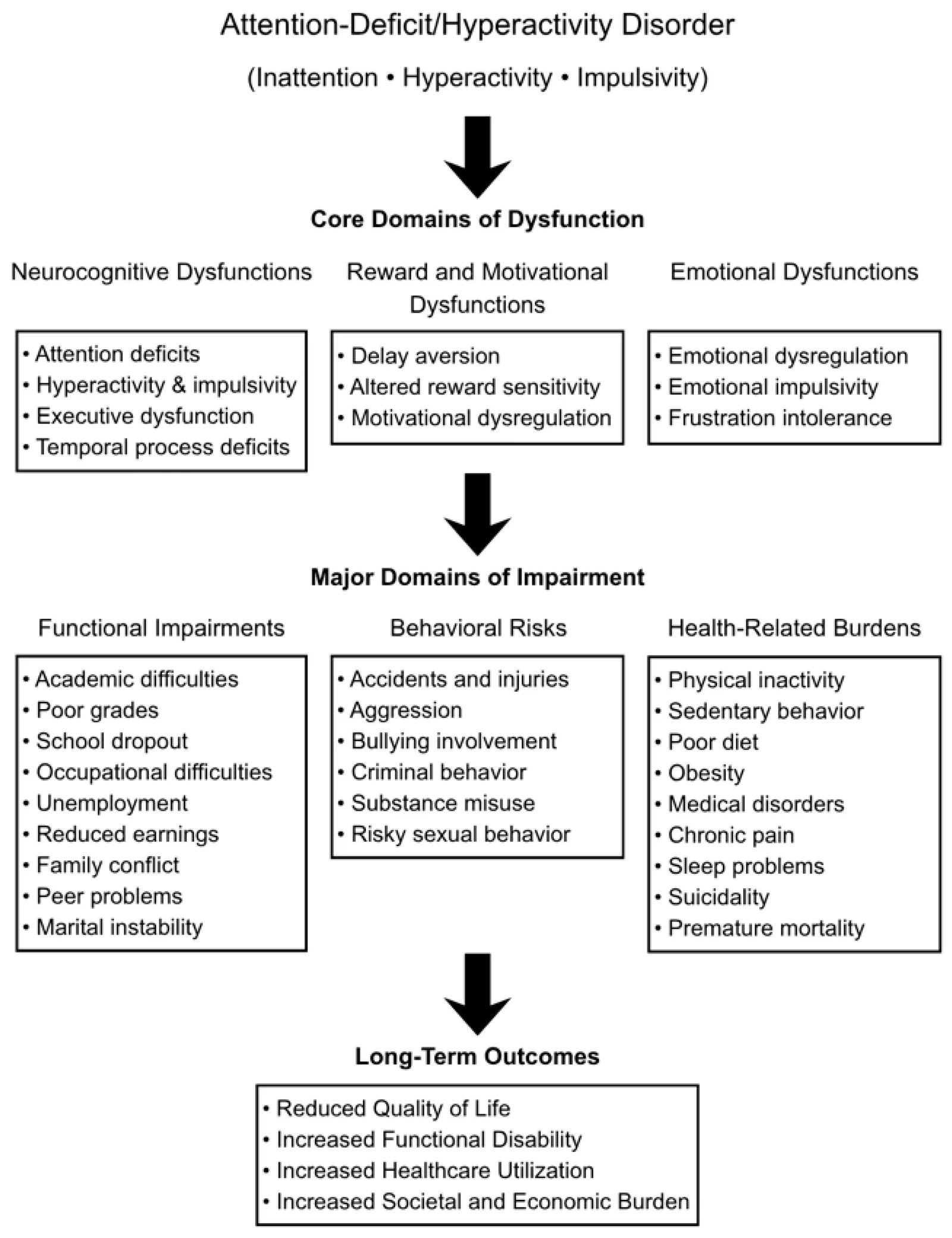
Conceptual framework illustrating how core ADHD symptoms may contribute to neurocognitive, reward/motivational, and emotional dysfunctions, which in turn are associated with functional impairments, behavioral risks, health-related burdens, and adverse long-term outcomes across the lifespan.

The evidence reviewed in this paper demonstrates that ADHD is associated with pervasive functional, psychosocial, and health burdens that extend far beyond its core signs and symptoms (Figure 1). These burdens span academic and occupational functioning, interpersonal relationships, injury risk, aggression and bullying, criminality, lifestyle-related and physical health outcomes, sexual risk behaviors, SUDs, self-harm, and premature mortality. Taken together, the evidence suggests that the magnitude and expression of these adverse outcomes may be shaped not only by ADHD itself but also by psychiatric comorbidities, sleep disturbances, environmental and socioeconomic adversity, medication use, lifestyle behaviors, and sex-specific vulnerabilities, particularly among females [189]. These interacting factors should be considered when interpreting the broader functional and health burden associated with ADHD.

Future research should prioritize longitudinal, mechanistic, and sex-stratified studies to clarify the causal pathways linking ADHD with adverse functional and health outcomes, including the roles of medication use, executive dysfunction, emotional dysregulation, reward and motivational processing, and lifestyle factors such as diet, physical activity, and screen time. Future studies should also investigate protective and resilience-related factors, such as sense of coherence (SOC), which has been associated with lower levels of delinquency, substance use, emotional distress, and functional impairment, as well as greater well-being among individuals with ADHD [190]. A better understanding of such protective factors may help identify individuals who are more resilient to adverse outcomes and inform the development of strengths-based interventions aimed at improving long-term functioning and quality of life. Clinically, these findings underscore the importance of early identification and timely access to evidence-based interventions aimed at reducing functional impairments and improving long-term outcomes across the lifespan [11,191,192,193]. Public health strategies that increase access to diagnosis and evidence-based treatments, reduce stigma, and address social determinants of health will be essential to mitigating the substantial individual, familial, and societal burden associated with ADHD.

## Figures and Tables

**Table 1:** Summary of Major Domains of Impairment Associated with ADHD.

Domain	Major Findings	Representative Evidence
Academic	Lower achievement, school dropout, reduced educational attainment	Massetti et al. (2008) [14]; Tamm et al. (2021) [15]
Occupational	Unemployment, reduced productivity, burnout, lower earnings	Knapp et al. (2011) [24]; Gjervan et al. (2012) [21]; Kessler et al. (2009) [26]; Fuermaier et al. (2021) [20]
Relationships	Family conflict, peer difficulties, marital dissatisfaction	Chang and Gau (2017) [29]; Hoza et al. (2005) [35]; Wymbs et al. (2021) [41]; Soares et al. (2021) [43]
Injuries	Vehicular accidents, sports injuries, mild traumatic brain injury	Barkley (2014) [50]; Ruiz-Goikoetxea et al. (2018) [51]; Brunkhorst-Kanaan et al. (2021) [49]; Adeyemo et al. (2014) [58]
Aggression/Bullying	Increased aggression, victimization and perpetration	Jensen et al. (2007) [60]; Speyer et al. (2022) [62]; Cuba Bustinza et al. (2022) [69]; Mayes et al. (2015) [71]
Criminality	Increased arrests, convictions, incarceration	Satterfield et al. (2007) [76]; Lundström et al. (2014) [78]; Mohr-Jensen et al. (2016, 2019) [79,80]
Physical Health	Obesity, cardiovascular disease, chronic pain	Cortese et al. (2016) [124]; Li et al. (2023) [142]; Pan et al. (2022) [136]; Mangerud et al. (2013) [138]
Substance Use	Increased substance use disorder risk	Charach et al. (2011) [164]; Lee et al. (2011) [165]; Wilens et al. (2011) [166]
Sexual Health	Risky sexual behavior, sexually transmitted infections, early pregnancy	Owens et al. (2017) [87]; Chen et al. (2018) [172]; Herweijer et al. (2024) [173]
Mortality	Reduced life expectancy, increased premature mortality	Dalsgaard et al. (2015) [176]; Barkley and Fischer (2019) [177]; O’Nions et al. (2025) [178]

## Data Availability

No new data were created or analyzed in this study. Data sharing is not applicable to this article.
